# Assessment of medical students' knowledge of primary limb sarcomas

**DOI:** 10.1186/s12909-024-05111-z

**Published:** 2024-02-13

**Authors:** Pedro Alcântara Botelho Machado, Gabriella Freitas Pereira Bartolomeu, Alycia Madureira Handeri, Maria Olívia Teixeira Silva, Ariel E. Hirsch, Ana Paula Drummond-Lage

**Affiliations:** 1 Post Graduation Department, Faculdade de Ciências Médicas de Minas Gerais, Belo Horizonte, Brazil; 2 School of Medicine, Faculdade de Ciências Médicas de Minas Gerais, Belo Horizonte, Brazil; 3 Boston University Chobanian & Avedisian School of Medicine, Boston, Massachusetts USA

**Keywords:** Sarcoma, Neoplasms, Medical education, Public health

## Abstract

**Introduction:**

Typically, oncology is not a structured part of the curriculum in Brazilian medical schools. Furthermore, sarcomas, which are uncommon tumors, are seldom covered in depth. A lack of comprehensive education on sarcomas might result in medical professionals being ill-equipped to care for patients with this condition.

**Objectives:**

To assess medical students' understanding and awareness of sarcomas and the specific principles related to these tumors.

**Materials and methods:**

A quantitative, cross-sectional study was conducted using a questionnaire, applied to medical students, focusing on the epidemiology, pathophysiology, and treatments of bone and soft tissue sarcomas. In all tests, the significance level adopted was 5%. The SPSS version 25.0 software was used.

**Results:**

Of the 825 questionnaires distributed, 325 were returned. Educational sessions on sarcomas did not appear to significantly improve the student's knowledge. Only 29.5% of students identified the lack of pain as an indicator of potential malignancy in soft tissue sarcomas, while 73.8% correctly recognized pain as a symptom of bone sarcomas. Limb amputation as the optimal surgical method for patient recovery was incorrectly reported by 39.1% of the sample.

**Conclusion:**

A great part of the surveyed population does not have adequate knowledge about the basic concepts associated with limb sarcomas. The minority of them are satisfied with the knowledge gained during their medical education about these tumors. Inadequate medical academic training may initially lead to the wrong clinical management of patients with bone and soft tissue tumor lesions. An educational effort is needed to enhance oncology education for medical students, especially concerning sarcomas.

## Introduction

Cancer is an important cause of mortality and morbidity and is expected to be the main cause of death in Brazil by 2030 [1]. Globally, neoplasms led to the death of about 9.9 million people in 2020 [2]. Oncology as a subject is not well represented in the undergraduate medical curriculum in Brazil, where there is no mandatory oncology training [3].

Among the different types of cancer, sarcomas are neoplasms originating from tissues of mesenchymal embryological origin and can arise in connective tissues such as bones, tendons, muscles, cartilage, as well as nerves, and blood vessels [4]. Didactically, sarcomas are divided into soft tissue sarcomas and bone sarcomas [5]. Around 50% of cases of sarcomas are located in the extremities (upper and lower limbs), including the pelvic and shoulder girdles. Other locations include the axial skeleton, trunk, and, in the case of soft tissue sarcomas, the retroperitoneum [6]. Soft tissue and bone sarcomas account for less than 1% of all neoplasms, being rare and heterogenous with various subtypes having a different prognostic. Furthermore, they may represent up to about 20% of solid neoplasms in the pediatric population [4]. They present significant lethality and, together, have a mean survival of 66% in 5 years [7].

Early detection of cancer offers higher chances of cure, and primary care physicians must be able to contextualize clinical findings, such as age, sex, association of symptoms, time of evolution, and other data, for appropriate diagnosis and management of patients [8].

The existing body of literature on the understanding of primary limb sarcomas among medical students is scarce. In a prior study, our group highlighted that medical students exhibit challenges in accurately interpreting images of bone sarcomas, and concluded that there is a crucial need to advance undergraduate education in oncology overall, with a specific emphasis on addressing sarcomas within this educational framework [9]

In this context, this study aimed to assess medical students' knowledge of oncological concepts related to bone and soft tissue sarcomas.

## Material and methods

A cross-sectional, quantitative, and exploratory study was carried out using a self-administered questionnaire developed by the researchers containing 20 multiple-choice questions, addressing academic knowledge about extremity sarcomas, including epidemiology, symptoms, physical examination, staging, and treatments of these tumors.

The questionnaire was distributed electronically to students from the 8th to the 12th graduation period, that is, those who have already attended the theoretical cycle and are starting or have already started internships at a private medical school in Belo Horizonte, Brazil.

The study was approved by the Faculdade de Ciências Médicas de Minas Gerais Research Ethics Committee (CAAE nº 48344021.6.0000.5134) and all participants previously signed a written informed consent form (ICF).

Data were presented in frequency tables with absolute frequencies and their respective percentages. Categorical variables were compared using the Chi-square test or Fisher's exact test. In all tests, the significance level adopted was 5%. The software used for the analysis was SPSS version 25.0.

## Results

The sample consisted of 325 participants, mostly female (64.3%), mean age of 23.18±2.54 years, ranging from 20 to 40 years. No participant reported having taken a mandatory course in oncology, and 59.7% responded that they had received an academic approach to sarcomas distributed in different disciplines, such as pathology (49.5%), internal medicine (38.1%), surgery (12.9 %), orthopedics (10.8%), pediatrics (9.8%) and radiology (4.6%).

Research participants were stratified into two groups: 1) the first one received a didactic approach about sarcomas (59.7%), and 2) the second did not receive it (40.3%), to assess the impact of the didactic approach on the knowledge acquired during graduation.

When evaluating the perception of knowledge acquired about bone sarcomas during graduation, it was observed that the didactic approach resulted in greater knowledge only about epidemiology (*p*= 0.011). The number of students who considered the acquired knowledge sufficient was low, ranging from 1.2% (chemotherapy treatment) to 19.1% (pathology). In the assessment of symptoms, participants from both groups mentioned symptoms unrelated to bone tumors in the initial phase, with statistical differences for palpable mass (*p* = 0.044), edema in the limbs (*p*=0.026), and fatigue (*p*=0.011). Limb weakness was misquoted by 74.1% of the study population (Table 1).

**Table 1:** Self-perception of bone sarcomas knowledge according to the presence of a didactic approach in medical school (*n*=325)

Do you consider your knowledge sufficient regarding this?	Didactic approach to sarcomas		Total	*p*-value
**Specific concepts**	**No N (%)**	**Yes N (%)**	**N (%)**	
Epidemiology	5 (3.8)	23 (11.9)	28 (8.6)	**0.011**^**a**^
History/physical examination	16 (12.2)	33 (17.0)	49 (15.1)	0.236^a^
Pathology	28 (21.4)	34 (17.5)	62 (19.1)	0.386^a^
Staging	10 (7.6)	17 (8.8)	27 (8.3)	0.717^a^
Chemotherapy	1 (0.08)	3 (1.5)	4 (1.2)	0.651 ^b^
Radiotherapy	1 (0.08)	4 (2.1)	5 (1.5)	0.418 ^b^
Surgery	6 (4.6)	12 (6.2)	18 (5.5)	0.535^a^
Image	15 (11.5)	24 (12.4)	39 (12)	0.802^a^
**Symptoms**				
Fever	43 (32.8)	57 (29.4)	100 (30.8)	0.509^a^
Local pain	114 (87.0)	156 (80.4)	270 (83.0)	0.119^a^
Palpable mass	39 (29.8)	79 (40.7)	118 (36.3)	**0.044**^**a**^
Weight loss	115 (87.8)	160 (82.5)	275 (84.6)	0.193^a^
Edema in limbs^c^	42 (32.1)	86 (44.3)	128 (39.4)	**0.026**^**a**^
Weakness in limbs^c^	98 (74.8)	143 (73.7)	241 (74.1)	0.825^a^
Fatigue^c^	104 (79.4)	129 (66.5)	233 (71.7)	**0.011**^**a**^
Local ulceration^c^	18 (13.7)	32 (16.5)	50 (15.4)	0.500^a^
Local lymphadenopathy^c^	68 (51.9)	99 (51.0)	167 (51.4)	0.877^a^

^a^Chi-square test

^b^Fisher's test

^c^incorrect answers

When the same evaluation was carried out concerning soft tissue sarcomas of the extremities, it was observed that the didactic approach contributed to the perception that knowledge was sufficient for epidemiology (*p*= 0.011) and anamnesis (*p* = 0.026), and for the evaluation of the symptoms fever (*p* = 0.027) and local pain (*p* = 0.021). The number of students who considered the acquired knowledge sufficient ranged from 0.9 (radiotherapy) to 14.1% (pathology). Regarding the risks of malignancy, the absence of pain was most cited by students who received a didactic approach about sarcomas (*p*=0.008), and 31.7% of the sample erroneously cited the location of the lesion in the subcutaneous tissue as a suggestion of malignancy (Table 2).

**Table 2:** Self-perception of sarcomas of soft tissues and extremities knowledge according to the presence of a didactic approach in medical school (*n*=325)

Do you consider your knowledge sufficient regarding this?	Didactic approach to sarcomas		Total	*p*-value
**Specific concepts**	**No N (%)**	**Yes N(%)**	**N (%)**	
Epidemiology	5 (3.8)	23 (11.9)	28 (8.6)	**0.011**^**a**^
History/physical examination	11 (8.4)	33 (17.0)	44 (13.5)	**0.026**^**a**^
Pathology	21 (16.0)	25 (12.9)	46 (14.1)	0.425^a^
Staging	4 (3.1)	9 (4.6)	13 (4)	0.474^a^
Chemotherapy	2 (1.5)	2 (1.0)	4 (1.2)	1,000^b^
Radiotherapy	0 (0.0)	3 (1.5)	3 (0.9)	0.276^b^
Surgery	1 (0.8)	10 (5.2)	11 (3.4)	0.055^b^
Image	5 (3.8)	16 (8.2)	21 (6.5)	0.111^a^
**Symptoms Knowledge**				
Fever	37 (28.2)	78 (40.2)	115 (61)	**0.027**^**a**^
Weight loss	97 (74.0)	151 (77.8)	245 (75.4)	0.431^a^
Palpable mass	107 (81.7)	141 (72.7)	248 (76.3)	0.061^a^
Edema in limbs*	80 (61.1)	133 (68.6)	213 (65.5)	0.164^a^
Weakness in limbs*	72 (55.0)	99 (51.0)	171 (52.6)	0.486^a^
Local pain*	62 (47.3)	117 (60.3)	179 (55.1)	**0.021**^**a**^
Fatigue^c^	82 (62.6)	128 (66.0)	210 (64.6)	0.531^a^
Local ulceration^c^	80 (61.1)	111 (57.2)	191 (58.8)	0.489^a^
Local lymphadenopathy^c^	88 (67.2)	136 (70.1)	224 (68.9)	0.576^a^
**Malignancy suggestion**				
Location in the subcutaneous tissue^c^	45 (34.4)	58 (29.9)	103 (31.7)	0.397^a^
Location deep to the subcutaneous tissue	113 (86.3)	165 (85.1)	278 (85.5)	0.761^a^
Absence of pain	28 (21.4)	68 (35.1)	96 (29.5)	**0.008**^**a**^
6 cm lesion	114 (87.0)	160 (82.5)	274 (84.3)	0.269^a^
Progressive growth	114 (87.0)	167 (86.1)	281 (86.5)	0.808^a^

^a^Chi-square test

^b^Fisher's test

^c^incorrect answers

The results in Table 3 address concepts about sarcomas from the basic to the surgical area. Eight known wrong statements about sarcomas were asked and asked if they were True or false. There was no statistically significant difference in the correct answers of the participants when stratified between those who received or did not receive a didactic approach in the medical course, except when asked about limb amputation when the group that received an academic approach was more assertive (68.7% vs. 55.7%, *p*=0.018) (Table 3).

**Table 3:** Assessment of knowledge about sarcomas in general according to having received a didactic approach in medical graduation

Knowledge regarding sarcomas in general	Didactic approach on sarcomas.		Total	*p*-value*
	**Yes N(%)**	**No N (%)**	**N (%)**	
**Epidemiology**				
Soft tissue sarcomas are more common in children than in adults.				
True	71 (54.2)	107 (55.2)	178 (54.7%)	0.865
False	87 (44.8)	60 (45.8)	147 (45.3%)	
Bone sarcomas are more common in adults than in children.				
True	71 (36.6)	60 (45.8)	131 (40.3%)	0.097
False	71 (54.2)	123 (63.4)	194 (59.7%)	
Most bone sarcomas, even diagnosed in advanced stages, are curable.				
True	49 (25.3)	38 (29)	87 (26.7%)	0.454
False	145 (74.7)	93 (71)	238 (73.2%)	
Bone and soft tissue sarcomas are common neoplasms as well as those of the breast, prostate, lung, or intestine.				
True	53 (27.3)	28 (21.4)	81 (24.9%)	0.224
False	141 (72.7)	103 (78.6)	244 (75.1%)	
**Surgical treatment**				
Intralesional curettage offers a high chance of cure and a low rate of local recurrence.				
True	56 (28.9)	43 (32.8)	99 (30.6%)	
False	137 (70.6)	88 (67.2)	225 (69.4%)	0.546
Ulcerated tumors or pathological fractures do not interfere with the decision between amputation or surgery to preserve the limb				
True	31 (16.0)	24 (18.3)	55 (16.9%)	0.581
False	163 (84.0)	107 (81.7)	270 (83.1%)	
The surgical access routes recommended for performing the biopsy are preferably through the intermuscular septa and the neurovascular spaces				
True	132 (68.0)	87 (66.4)	219 (67.4%)	0.759
False	132 (68.0)	87 (66.4)	106 (32.6%)	
Amputation is the best treatment than preservation of the limb considering the proposed curative surgery.				
True	86 (44.3)	41 (31.3)	127 (39.1%)	**0.018**
False	108 (55.7)	90 (68.7)	198 (60.9%)	

^*^Chi-square test

Most participants answered correctly about the age group with the highest prevalence of bone sarcomas (59.7%), the prevalence of sarcomas in general (75.1%), as well as the concept that the progression of the disease decreases the chance of cure (73.2%), and on the indication of intralesional curettage (69.4%). The presence of a pathological fracture or local ulceration was correctly evaluated by 83.1% as factors that worsen the prognosis of limb preservation. The majority of participants (60.9%) understood the concept that amputation is not necessarily the best treatment when the aim is to cure the disease. However, there was a smaller number of correct answers regarding the age group with the highest incidence of soft tissue sarcomas (45.3%) and the ideal location for sarcoma biopsies (32.6%) (Table 3).

Among the study participants, only 1.5% stated that they were satisfied with the knowledge acquired about sarcomas during graduation. The remainder reported wanting to take an elective or mandatory course in cancerology during graduation (32.3% and 28.9% respectively) or a course specifically addressing sarcomas (4%). Other participants (31.4%) responded that they would have liked to have received more information about sarcomas during graduation within the existing disciplines in the curriculum.

Only 1.8% said they did not consider sarcomas important or prevalent to deserve attention in the curriculum.

## Discussion

### Oncology at medical school

In Brazil, 58.7% of medical schools do not offer a specific discipline addressing cancerology and, when offered, it is not always mandatory [10]. The institution of this study also does not offer a discipline of cancerology, either mandatory or optional. The non-systematized and fragmented teaching pattern on cancerology in the medical course was confirmed in this work, where it was observed that sarcomas were addressed in six different disciplines. In this study, 40.3% of students reported not having a didactic approach to sarcomas. The variation in the level of knowledge of students from the same institution raises hypotheses on teachers' autonomy in whether or not to address the topic, or perhaps the students themselves, because they do not have a systematized content, do not remember that they studied sarcomas.

This study showed that a low percentage of students consider their knowledge of specific topics to be sufficient (epidemiology, anamnesis, physical examination, pathology, staging, chemotherapy, radiotherapy, surgery, and imaging) related to both bone sarcomas and soft tissue sarcomas. This data from the student's perspective corroborates the work carried out by Neeley et al. [11] who reported the low confidence of students regarding oncology in general, including sarcomas.

Sarcomas corresponds to about 1% of all neoplasms [12]. Most of the study population (75.1%) knows that these neoplasms are rarer than others, such as the breast, prostate, lung, and intestine, which corresponded, respectively, to 11.7%, 7.3%, 11.4%, and 10.0% of all cancer cases in the world in 2020 [13].

### Bone sarcomas

About 3000 bone sarcomas are annually diagnosed in the United States. The most common types are osteosarcoma, Ewing's sarcoma, and chondrosarcoma. Bone sarcomas have a bimodal distribution with peaks between the first and second decade of life and during the seventh decade. In general, they are more frequent in children than adults [14]. This epidemiological aspect was known by 59.7% of the participants in the present study.

Among the specific symptoms of bone sarcomas, early progressive, non-mechanical pain is the main initial symptom, and, in this context, subsequent investigation with an imaging method is mandatory [15]. For pain, 83% of students correctly considered it as a semiological characteristic related to bone sarcomas, which may contribute to a more agile and appropriate referral of the case.

Besides, local ulceration and satellite lymphadenopathy are not classically described as initial symptoms of bone sarcomas [16]. In the present study, there was heterogeneity in the pattern of responses referring to symptoms related to bone sarcomas, since more than 50% of participants mistakenly considered local lymphadenopathy as prevalent and more than 84% agreed that ulceration of these tumors is a rare event.

Some symptoms such as malaise, fever, involuntary weight loss, fatigue, and weakness are present in advanced stages in the vast majority of neoplasms [17]. However, in more advanced stages, bone sarcomas present specific symptoms, such as edema in the limbs and a palpable mass, when the tumor breaks through the cortical bone and distends the periosteum [15]. The population of this study considered fever to be less present in these patients and weight loss to be more frequent.

The progression of the disease directly interferes with the decrease in the chance of curing sarcomas. Osteosarcoma, the most common bone sarcoma, has a worse prognosis when diagnosed in patients with more than four weeks of symptoms. When there are lung metastases at diagnosis, the 5-year survival drops from 80 to 47% [18]. This concept of worsening outcomes with the presence of advanced disease was known by 73.2% of the participants.

### Soft tissue sarcomas

Soft tissue sarcomas affect patients over 40 years of age in more than 78% of cases. There are about 13,000 cases diagnosed per year in the United States and around 3,400 cases diagnosed per year in Brazil. In Brazil, there is probably underreporting due to poor reporting by the health system [19]. This epidemiological data was unknown by 54.7% of the participants.

Soft tissue tumors in the limbs are common, but only about 1 in 200 diagnoses is a malignancy. Pain is not found initially in most patients and may lead to an error of judgment with a presumption of benignity [20]. In this study, students overestimated pain for soft tissue sarcomas (55.1%), and those who had a didactic approach were the ones who most missed the concept (60.3%). The absence of pain was described as important for suspicion of soft tissue sarcomas by only 29.5%. Most instances of soft tissue sarcomas typically manifest as painless lumps, and individuals may not notice them until they significantly grow in size [21]. This result highlights the need for special focus on this aspect from the perspective of medical education, as the delay in diagnosing a sarcomatous lesion harms the patient's prognosis, as for each centimeter of growth, the patient may lose 3 to 5% of the chance of healing [22]. Classically, most soft tissue sarcomas are diagnosed with lesions larger than 5 cm, deep to the muscle fascia, and with progressive growth [20]. All these aspects were correctly interpreted by more than 80% of the students.

Again, local ulceration is not found in the early stages of soft tissue sarcomas, but satellite lymphadenopathy may be present in some types of these tumors [23]. This alteration was correctly described by 69.9% of the students.

### General concepts about sarcomas

Adequate surgical margin is an important tool for limb prognosis and improved survival of patients with sarcomas. Enneking et al. [24] proposed a system of surgical margins for musculoskeletal tumors. Appropriate margins would be the wide type, where the tumor is resected with an adjacent normal tissue layer, and the radical type, in which the neoplasm is resected together with its entire native anatomical compartment, for example, a muscle group. Other types of margins, such as the intralesional and marginal, violate the lesion compartment and are inadequate for sarcoma surgeries [25]. Intralesional curettage was correctly judged as incorrect in sarcoma surgeries by 69.4% of the students, demonstrating that most of them know this surgical concept correctly.

In this study, 67.4% of the participants missed the question that addresses knowledge about biopsies. Biopsy, an important tool for the diagnosis and staging of sarcomas, must also follow principles for its execution. They should not be performed between the muscular septa or through the neurovascular spaces, since there is a risk of contamination of other anatomical compartments and a consequent decrease in the chance of saving the limb. It should be performed in such a way that only the tumor compartment is violated, that hematoma formation is avoided, and that the biopsy path can be resected in the definitive surgery, as it is potentially contaminated by tumor cells [26]. Because it is a necessary procedure for diagnosis, it should be better addressed in the medical curriculum.

The association between ulceration and pathological fracture as a relevant factor for the decision to save the limb was correctly answered by 83.1% of the participants. Ulcerated sarcomas and/or pathological fracture indicate local progression of the disease and tumor extravasation to another anatomical compartment, be the cortical bone or the skin. Limb-preserving surgery is the gold standard for the resection of soft tissue sarcomas, but the presence of local ulceration worsens the limb prognosis and can lead to amputation rates of 13 to 35% [27]. For bone sarcomas, the presence of a pathological invoice forms a local hematoma contaminated by tumor cells that seems to spread the tumor to adjacent tissues. This context significantly increases the indication of amputation of a limb instead of preservation surgery as a way to improve the surgical margin and avoid local recurrence [28].

For 60.9% of the students, the ablative treatment (amputation) is not superior to limb preservation surgery in terms of the cure outcome. The correct answer is that the surgical decision between amputating or preserving the limb does not interfere with the cure rate of sarcomas as long as an adequate margin is obtained [29]. Amputations are even rare and indicated in less than 10% of cases in reference centers [30].

Around 5 to 31% of bone sarcomas and 18 to 66% of soft tissue sarcomas are initially approached surgically, even by general practitioners. These numbers have already been related to the failure of the educational system to bring relevant knowledge about sarcomas to future physicians [31].

In summary, the current didactic concept concerning sarcomas in the medical curriculum appears to be ineffective, and there are plausible explanations such as i) limited relevance, where stakeholders may not perceive sarcomas as highly pertinent to the broader medical curriculum or clinical practice [9]; ii) curricular overload as the existing medical curriculum is already dense, making the inclusion of sarcomas burdensome and potentially reducing student attention and retention [32]; iii) misalignment of assessment methods with learning objectives [33]; and iv) a lack of continuous feedback from students, educators, and healthcare professionals during curriculum implementation that could impact necessary adjustments for improvement [34].

Addressing these challenges requires a comprehensive curriculum evaluation involving diverse stakeholders and subsequent adjustments to teaching methods, assessment strategies, and the curriculum itself based on gathered feedback. Based on our findings we propose some strategies and suggestions to enhance oncology education in medical institutions: i) provide training and professional development opportunities for faculty members to stay updated on the latest advancements in oncology, ii) encourage collaboration with oncology specialists and researchers, iii) incorporate case-based learning and practical experiences to foster critical thinking skills and clinical reasoning through case studies, iv) facilitate early and regular clinical exposure to oncology patients through rotations, clerkships, and elective programs, and iv) keep the curriculum dynamic by regularly updating content to reflect emerging trends, new treatment modalities, and evolving research in oncology.

This study has some limitations: i) the limited generalizability as the findings may not be broadly applicable to other institutions or populations, as characteristics and perspectives may vary; ii) the limited variation in responses as medical students from a single institution may share similar educational backgrounds, exposure, or experiences, resulting in limited variation in survey responses; iii) the single-point-in-time snapshot as a survey conducted at a single institution captures data at a specific point in time, making it challenging to account for dynamic changes that may occur over time.

## Conclusion

The results of the present study show that most students do not have adequate knowledge about the concepts in oncology related to sarcomas and that the current didactic approach to sarcomas in the curriculum did not seem to impact knowledge related to these neoplasms.

The lack of a systematized curriculum that includes the most important topics of knowledge on the subject has contributed to this pattern of results. Strategies for optimizing teaching in oncology contemplating sarcomas must be sought to deliver a better line of care for the patients. In this context, the recommended hours for sarcoma teaching in the medical curriculum should consider factors such as the overall curriculum structure, the importance of sarcomas in oncology, and specific learning objectives. It is recommended that a comprehensive and mandatory module in Basic Oncology be incorporated into the curriculum, encompassing a thorough exploration of sarcomas. Recognizing the constraints of time in medical education and the imperative for a well-rounded oncology curriculum, we propose a focused approach that emphasizes fundamental aspects of sarcomas, such as their clinical presentation, epidemiology, and initial imaging evaluation. This knowledge is deemed congruent with the training requirements for future general practitioners. To optimize learning outcomes, a blend of didactic lectures and interactive methods, such as case studies and clinical exposure, is advised. A suggested timeframe of 1 to 3 hours within the course is deemed adequate for this targeted approach. Furthermore, it is emphasized that regular updates, both theoretical and practical, reflecting advancements in sarcoma research and clinical practice, should be integrated to ensure the curriculum remains current and aligns with evolving medical knowledge.

Based on study´s findings, there are several directions for further research: i) assessment of specific sarcoma knowledge gaps, ii) investigation into pedagogical approaches, iii) comparison across Medical Institutions, iv) impact of education on clinical decision-making, and v) development and evaluation of educational interventions.

## Data Availability

The datasets used and analyzed during the current study are available from the corresponding author upon reasonable request.
